# Collateral impact of COVID-19: why should children continue to suffer?

**DOI:** 10.1007/s00431-021-03963-x

**Published:** 2021-02-13

**Authors:** Prasad Nagakumar, Ceri-Louise Chadwick, Andrew Bush, Atul Gupta

**Affiliations:** 1 Paediatric Respiratory Medicine, Department of Paediatric Respiratory Medicine, Birmingham Women’s and Children’s Hospital NHS Foundation Trust, Steelhouse Lane, Birmingham, B4 6NH UK; 2 Birmingham Acute Care Research, Institute of Inflammation and Aging, University of Birmingham, Birmingham, UK; 3 Paediatric Respiratory Registrar, Department of Paediatric Respiratory Medicine, Birmingham Women’s and Children’s Hospital NHS Foundation Trust, Steelhouse Lane, Birmingham, B4 6NH UK; 4 Paediatrics and Paediatric Respirology, Imperial College & Consultant Paediatric Chest Physician, Royal Brompton Harefield NHS Foundation Trust, Sydney Street, London, SW3 6NP UK; 5 Paediatric Respiratory Medicine, Department of Paediatric Respiratory Medicine, King’s College Hospital, London & King’s College London, Denmark Hill, London, SE5 9RS UK; 6 Institute for Women’s and Children’s Health, King’s College London, London, UK

**Keywords:** COVID-19, Children and young people, Vaccine, Mental health

## Abstract

The COVID-19 pandemic caused by SARS-COV-2 virus fortunately resulted in few children suffering from severe disease. However, the collateral effects on the COVID-19 pandemic appear to have had significant detrimental effects on children affected and young people. There are also some positive impacts in the form of reduced prevalence of viral bronchiolitis. The new strain of SARS-COV-2 identified recently in the UK appears to have increased transmissibility to children. However, there are no large vaccine trials set up in children to evaluate safety and efficacy. In this short communication, we review the collateral effects of COVID-19 pandemic in children and young people. We highlight the need for urgent strategies to mitigate the risks to children due to the COVID-19 pandemic.

**Table:** 

**What is Known:** • *Children and young people account for <2% of all COVID-19 hospital admissions* • *The collateral impact of COVID-19 pandemic on children and young people is devastating* • *Significant reduction in influenza and respiratory syncytial virus (RSV) infection in the southern hemisphere*	
**What is New:** • *The public health measures to reduce COVID-19 infection may have also resulted in near elimination of influenza and RSV infections across the globe* • *A COVID-19 vaccine has been licensed for adults. However, large scale vaccine studies are yet to be initiated although there is emerging evidence of the new SARS-COV-2 strain spreading more rapidly though young people.* • *Children and young people continue to bear the collateral effects of COVID-19 pandemic*

**What is Known:**

• *Children and young people account for <2% of all COVID-19 hospital admissions*

• *The collateral impact of COVID-19 pandemic on children and young people is devastating*

• *Significant reduction in influenza and respiratory syncytial virus (RSV) infection in the southern hemisphere*

**What is New:**

• *The public health measures to reduce COVID-19 infection may have also resulted in near elimination of influenza and RSV infections across the globe*

• *A COVID-19 vaccine has been licensed for adults. However, large scale vaccine studies are yet to be initiated although there is emerging evidence of the new SARS-COV-2 strain spreading more rapidly though young people.*

• *Children and young people continue to bear the collateral effects of COVID-19 pandemic*

Children and young people (CYP) account for <2% of the COVID-19 infections caused by the SARS-CoV-2 virus [1]. Although the numbers are smaller than in adults, severe COVID-19 and deaths have been noted in children with COVID-19 infection [2]. Long-term consequences of COVID-19 have also been reported in CYP. Also, the Paediatric Multisystem Inflammatory Syndrome Temporally associated with COVID-19 (PIMS-TS) has resulted in some very sick CYP being admitted to intensive care units. As the pandemic continues, wider direct and collateral effects are becoming apparent. The collateral effects of COVID-19 have resulted in devastating impacts on children’s health and well-being due to missed education, healthcare delivery, mental health, and social consequences [3].

## Impact on winter respiratory viruses—a silver lining of the COVID-19 pandemic

Lower respiratory tract infections are a leading cause of morbidity and mortality around the world. There are more than 2.38 million deaths a year from pneumonia or bronchiolitis making it the sixth leading cause of mortality in all ages and the leading cause amongst those under 5 [4]. Viral respiratory illnesses result in a large proportion of high dependency and intensive care admissions during the winter months and those with underlying medical conditions are particularly vulnerable. There are minimal existing measures to prevent such viral infections, apart from palivizumab (not licensed over age 2 years and only given to high-risk children, who actually are the minority of those admitted) influenza vaccines and Tamiflu.

**Fig. 1:**
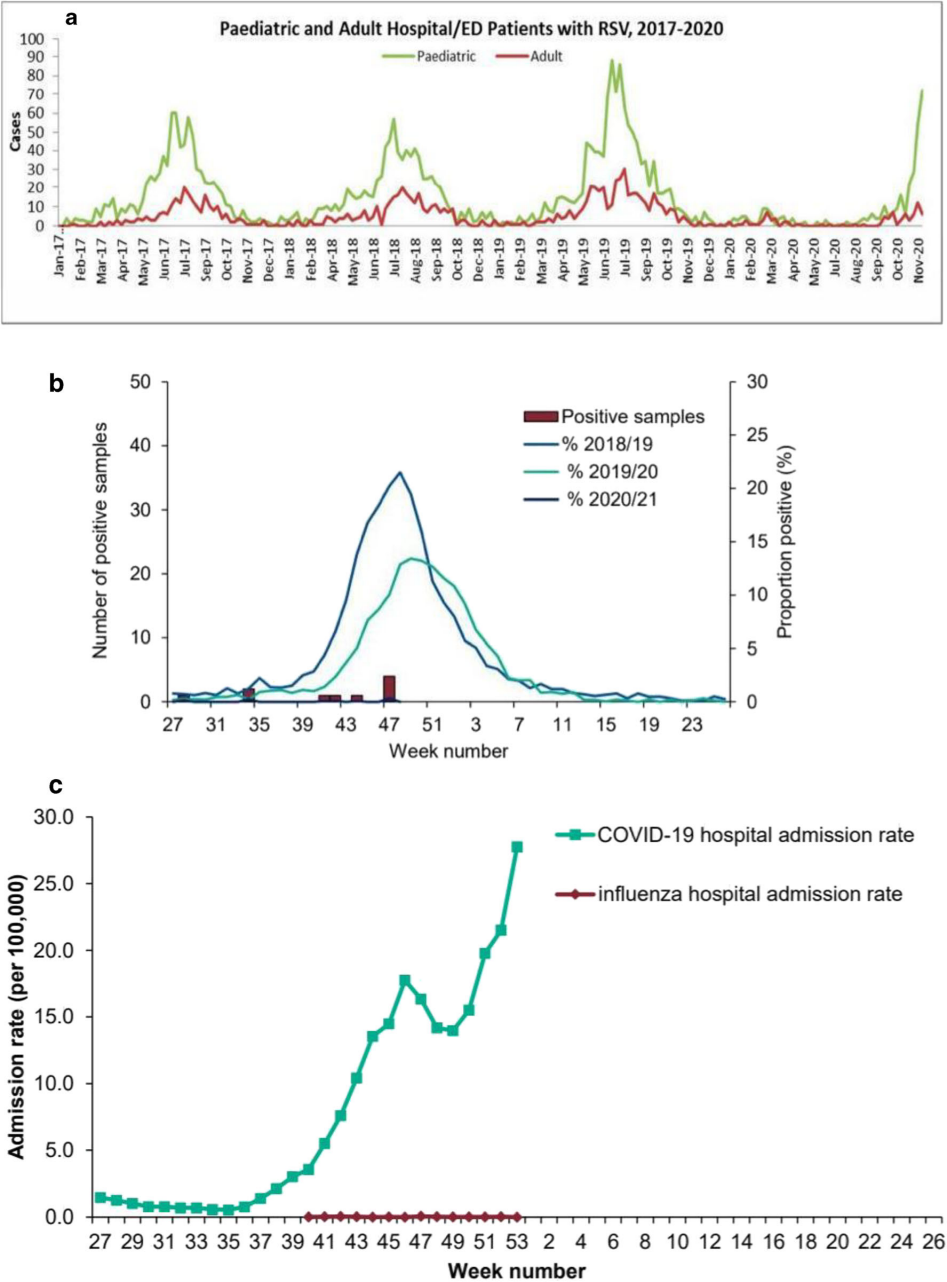
**a** Time series data from Western Australia showing significant reduction in respiratory syncytial virus infection in children and adults in 2020 winter (June–August) but increased infection rate in spring (September–November). **b** No influenza cases in England in end of November (week 48, brown bars). Data form the Public Health England national Influenza and COVID19 surveillance report comparing data from 2020 to 2019 and 2018. Data includes both children and adults. **c** No influenza hospital admissions in England in December 2020 despite significant COVID-19 hospital admission rate. Data from the Public Health England surveillance report includes both adults and children

The incidence of RSV and influenza in children and young people was significantly reduced during 2020. Southern hemisphere data shows a reduction in peak incidence of RSV from around 30% to zero [5] (Fig. 1a). Public Health England data shows a similar reduction in the UK with influenza reduced by 90% and RSV infection rates also close to zero [6] (Fig. 1b) in children and adults. There may be a later peak incidence this year but early data are encouraging that this will be significantly lower than previous years (Fig. 1a).

Reduced social interaction, frequent hand washing, the use of facemasks in public places, and reduced atmospheric pollution may have reduced winter virus infection rates [7]. Are public health measures responsible for the impressive reduction in viral LRTI and infection irrespective of geography, socio-economic status, and comorbidities? It is intriguing to note that despite increased isolation of SARS-COV-2 in children during December in England, the rates of RSV and influenza infection rates remained extremely low [8] (Fig. 1c). Whatever the cause of such significant reduction, there are important lessons to learn for the future. Will the policymakers, politicians, and the general public be willing to continue these public health measures following the COVID-19 pandemic? And if so, to what extent are these measures sustainable? If respiratory virus infections can be reduced, this will reduce demands on health services. The impact of reduced incidence of viral infections on viral wheeze and later asthma is yet to be quantified. Public awareness and legislation have resulted in significant changes in population behaviour and collateral beneficial effects of COVID on the impact of other winter viruses.

## Safeguarding and mental health implications

The effects of social distancing measures, chronic isolation, cessation of usual activities, and employment are expected to result in an increase in mental health disorders and substance misuse in adults. Furthermore, alcohol sales have increased by 31.4% and calls to the National Domestic Abuse Helpline have increased by approximately 50% since social distancing measures were introduced [9].

The Children’s Commissioner for England has highlighted the detrimental effect on the mental health of CYP. Changes to external support provided by health, social, and education services and isolation from support networks in wider society is to the detriment of identifying and monitoring CYP at risk of harm. These services need enhanced funding to support young people during these difficult times.

## Vaccine

Children and young people have been in the firing line of uncertainty throughout the COVID-19 pandemic. The UK has approved three vaccines effective against SARS-COV-2 for use in adults and more vaccines will likely follow soon. The COVID-19 vaccine studies in adults have moved at a lightning speed from trial set up to regulatory approval. Initial trials in 12–17-year-olds have commenced but the process for children and young people is lagging behind and there appears to be no perceived urgency in initiating vaccine studies in children. In contrast, there are 70 studies in adults still evaluating hydroxychloroquine in adult COVID-19 patients [10]. Children in secondary schools have been identified as a significant source for transmission of COVID-19 infection in the UK, probably related to the new variant known as B.1.1.7. The preliminary data show this variant to be associated with no increase in hospitalisations in children. Younger age groups have played an integral role in the success of previous vaccination programmes. The imperatives for immunising children include the prevention of severe forms of COVID (acute respiratory and PIMS-TS) and to prevent transmission both to other children and likely to adults.

It is important we act quickly with efficient and safe target groups to expand the use of vaccination and ensure that children and young people are not left behind. However, the safety of the vaccines in children should obviously be determined first.

We have an opportunity and a responsibility to act now and act effectively to avoid long-term disastrous and devastating effects on CYP health and welfare. The voice of CYP must be heard by the public and the policymakers and their education, mental well-being, and health should have the highest priority.

## Data Availability

N/A.

## Abbreviations

COVID-19: Coronavirus disease 2019
LRTI: Lower respiratory tract infection
CYP: Children and young people

## Code availability

N/A.
